# Body Mass Index, Waist Circumference, and Cognitive Decline Among Chinese Older Adults: A Nationwide Retrospective Cohort Study

**DOI:** 10.3389/fnagi.2022.737532

**Published:** 2022-03-07

**Authors:** Fang Liang, Jialin Fu, Justin B. Moore, Xinge Zhang, Yijia Xu, Nan Qiu, Yechuang Wang, Rui Li

**Affiliations:** ^1^School of Public Health, Wuhan University, Wuhan, China; ^2^Department of Implementation Science, Division of Public Health Sciences, Wake Forest School of Medicine, Winston-Salem, NC, United States; ^3^Department of Medicine and Therapeutics, The Chinese University of Hong Kong, Prince of Wales Hospital, Hong Kong, Hong Kong SAR, China; ^4^School of Nursing, Wuhan University, Wuhan, China

**Keywords:** body mass index, waist circumference, cognitive decline, older adults, cohort

## Abstract

**Background:**

The reported associations between body mass index (BMI), waist circumference (WC), and cognitive decline are not consistent, especially in older adults.

**Objective:**

This study aims to investigate the longitudinal associations of BMI, WC, and their change values with cognitive decline among Chinese adults aged 60 years and older and to examine the potential moderating effect of sex on these relationships.

**Methods:**

The participants in this study were from waves one to four (2011–2018) of the China Health and Retirement Longitudinal Study (CHARLS). Cognition function, BMI, and WC were measured at four examinations over 7 years. The interview-based cognitive assessments of memory, orientation and attention, and visuospatial ability were recorded. Standardized global cognitive scores were generated. BMI and WC were objectively measured. Mixed-effects models were performed to evaluate the associations.

**Results:**

A final sample of 3,035 Chinese older adults [mean (SD) age, 66.94 (5.43) years; 40.16% (*n* = 1,219) women] were included. Higher BMI (estimate = 0.0107; SE = 0.0024; *p* < 0.0001) and WC (estimate = 0.0019; SE = 0.0006; *p* = 0.0037) were associated with slower cognition score decline over a 7-year follow-up, while greater BMI variability (estimate = −0.0365; SE = 0.0116; *p* = 0.0017) was related to faster cognition score decline. The results were not modified by sex. BMI-defined overweight (estimate = 0.0094; SE = 0.0043; *p* = 0.0298) was associated with a slower cognition score decline, and both large weight gain (estimate = −0.0266; SE = 0.0074; *p* = 0.0003) and large WC loss (estimate = −0.0668; SE = 0.0329; *p* = 0.0426) were associated with faster cognition score decline.

**Conclusion:**

Among Chinese older adults, higher BMI, higher WC, and overweight are related to slower cognitive decline, while greater BMI variability, large weight gain, and large WC loss are associated with faster cognitive decline.

## Introduction

The global world is experiencing rising proportions of older adults (age = 60 years or above), and this proportion is anticipated to be >20% in 2050. China has the largest older population in the world, accounting for 201 million in 2015, and it is projected to increase to 479 million by 2050 (United Nations DESA)^1^. Concurrently, the number of elderly individuals with dementia is rising dramatically. A nationwide survey completed in 2018 estimated that 15 million Chinese older adults have dementia (Jia et al., 2020). The prevention of dementia is essential given that curative treatments or therapies for dementia lack effectiveness (Livingston et al., 2017). Accelerated cognitive decline has been regarded as the primary risk factor for the development of neurodegenerative disorders, including dementia (Yankner et al., 2008; Karr et al., 2018); thus, it is a high priority to identify modifiable risk factors for cognitive decline (Middleton and Yaffe, 2009).

Obesity is an emerging but complex risk factor for cognitive decline and dementia, especially in older adults (Dye et al., 2017). Some studies reported that excess obesity contributes to cognitive decline and dementia (Gunstad et al., 2010; Besser et al., 2014; West et al., 2017; Ntlholang et al., 2018; Zhang et al., 2018; Liu et al., 2019; Rubin et al., 2019) while accumulating epidemiological studies suggesting that there is an apparent protective effect of obesity on preventing cognitive decline and dementia in older adults (Atti et al., 2008; Hughes et al., 2009; Kerwin et al., 2010; Coin et al., 2012; Luchsinger et al., 2013; Tolppanen et al., 2014; Aslan et al., 2015; Kim et al., 2016, 2020; Deckers et al., 2017; Rodriguez-Fernandez et al., 2017; Arvanitakis et al., 2018; Michaud et al., 2018; Momtaz et al., 2018; Aiken-Morgan et al., 2020). Additionally, considering the effect of aging on fat distribution, the regional distribution of obesity, such as central or overall, may be an important consideration in the association of obesity and cognitive decline. A 2017 cohort study demonstrated that higher WC is related to the faster rate of cognitive decline, whereas BMI was not related to the rate of decline (West et al., 2017). Women and men exhibit significant differences in obesity and weight changes (Walsemann and Ailshire, 2011; Link and Reue, 2017), which may confound the association between obesity and cognitive decline. At present, only a few cross-sectional studies have explored the association of obesity and decline in cognitive function in Chinese older adults (Zhou et al., 2010; Zhang et al., 2018; Hou et al., 2019; Li et al., 2020), and we are unaware of any research that explored the interaction of dynamic change in obesity to cognitive decline while considering sex difference in a Chinese sample. This study proposes to fill these gaps by focusing on Chinese older adults, accounting for dynamic changes in BMI and WC, as well as testing interactions with sex.

Thus, we conducted this nationwide retrospective cohort study among Chinese older adults to examine (1) the longitudinal impact of BMI and WC on cognitive decline, (2) the association of changes in BMI or WC over several time points for each subject and cognitive decline, and (3) whether sex modifies the relationship between BMI, or WC, or BMI stability or WC stability, and cognitive decline.

## Methods

### Study Design and Participants

The China Health and Retirement Longitudinal Study (CHARLS) was a nationally representative longitudinal investigation of Chinese community-dwelling residents aged 45 years or above, which is publicly available at http://charls.pku.edu.cn. Using a stratified multistage probability-proportional-to-size random-cluster sampling method, 17,707 participants were selected at baseline in 2011. Respondents were periodically resurveyed in 2013 (wave 2), 2015 (wave 3), and 2018 (wave 4) using a face-to-face interview. Details of the CHARLS are available elsewhere (Zhao et al., 2014). We analyzed BMI, WC, and cognitive function data from adults who participated in the 2011 baseline survey and at least one follow-up conducted in 2013, 2015, or 2018.

**Figure 1:**
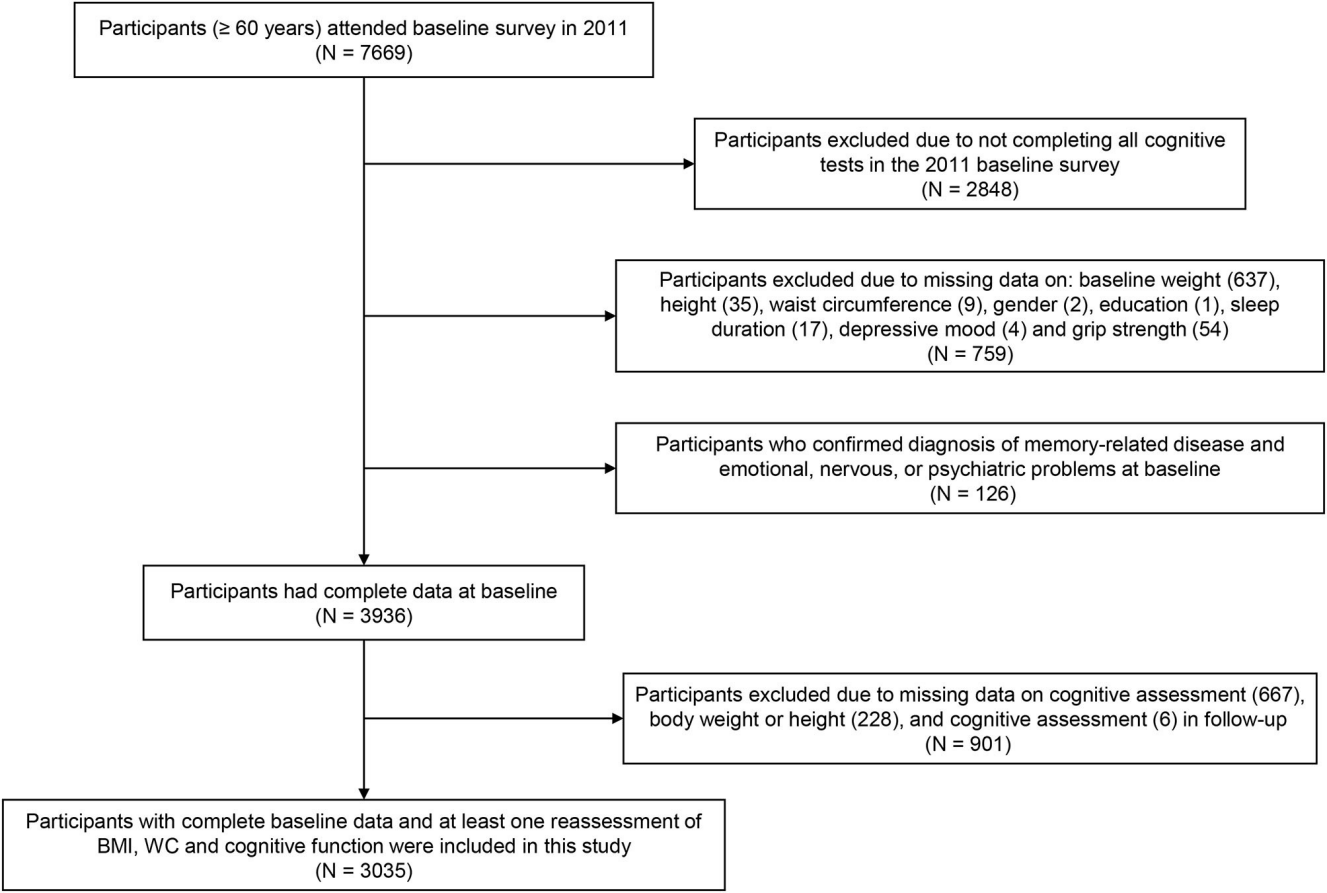
Flowchart of participants.

A total of 7,669 participants aged 60 years and older were recruited at baseline, while 2,848 individuals were excluded due to missing data on the cognitive assessment, and 759 participants were excluded due to lack of data at baseline on weight (637), height (35), WC (9), sex (2), education (1), sleep duration (17), depressive mood (4), or grip strength (54). Additionally, 126 respondents diagnosed with memory-related disease and emotional, nervous, or psychiatric problems at baseline were excluded. Similarly, 901 subjects with missing follow-up data on the cognitive assessment (667), body weight or height (228), and WC (6) were excluded, resulting in a sample of 3,035 Chinese older adults included in our analyses (Figure 1).

### Cognitive Assessment

Three domains of cognitive function, including memory, orientation and attention, and visuospatial ability, were measured in face-to-face interviews and in all waves.

#### Memory

The interviewers read out a set of 10 Chinese nouns at a slow steady rate and then asked the respondents to recall as many words as possible. The memory ability score was the average number of words that were successfully recollected immediately and at 4 min later. The resulting score ranged from 0 to 10 (Pan et al., 2018).

#### Orientation and Attention

Orientation and attention were appraised by the Mini-Mental State Examination (MMSE), which used items from the Telephone Interview of Cognitive Status (TICS-10; Knopman et al., 2010). Items included serial subtraction of 7 from 100 (up to 5 times), date (month, day, year), day of the week, and season of the year. The score was the aggregate number of correct answers and ranged from 0 to 10.

#### Visuospatial Ability

Visuospatial ability was evaluated by figure drawing. Participants were asked to observe a picture of 2 overlapping pentagons and draw a similar figure. The individual who successfully drew the picture got 1 point, and 0 point was given for an unsuccessful drawing (Chen et al., 2021).

#### Global Cognition

The global cognition score was computed as the sum of the memory, orientation and attention, and visuospatial scores, which ranged from 0 to 21. A lower score indicated worse cognitive performance.

A standardized *z*-score of global cognitive function was calculated to compare individual scores from different measurements according to the baseline mean and SD. Specifically, each score was standardized by subtracting the baseline mean and dividing it by the baseline SD. The change in cognitive function was defined as the follow-up *z*-scores minus the baseline *z*-score, and the rate of cognitive decline was calculated as the change in cognitive function divided by the follow-up wave.

### Body Mass Index (BMI) and Waist Circumference (WC)

Bodyweight, height, and waist circumference (WC) were objectively measured by trained investigators with standardized equipment. Bodyweight and height of participants were measured in light clothing with shoes off. WC was measured at a point midway between the lowest rib and the iliac crest in a horizontal plane using non-elastic tape. Body mass index (BMI) was calculated as weight in kilograms divided by the square of height in meters. According to the criteria of weight for Chinese adults, baseline BMI (kg/m^2^) was categorized as follows: underweight (BMI <18.5), normal (18.5 ≤ BMI <23.9), overweight (24 ≤ BMI <27.9), and obese (BMI ≥ 28) (Wu et al., 2021). Baseline WC (cm), as indicators of central adiposity, was classified into three categories: no central obesity (WC <85 for men and WC <80 for women), central pre-obesity (85 ≤ WC <90 for men and 80 ≤ WC <85 for women), and central obesity (90 ≤ WC for men and 85 ≤ WC for women; Jia et al., 2010).

We used the baseline information and up to three follow-up assessments to calculate person-specific BMI or WC range at each follow-up for each participant (Capuano et al., 2018; Aiken-Morgan et al., 2020). In addition, BMI and WC change were classified into 5 groups, namely, large loss (< -10%), small loss (−10 to −5%), stable (−5 to <5%), small gain (5 to <10%), and large gain (more than 10%; Newman et al., 2001; Wu et al., 2021).

### Covariates

The demographic characteristics included age, sex, and education. Lifestyle factors included smoking status (current = 2, former = 1, or never = 0), drinking status (current = 2, former = 1, or never = 0), and sleep duration. Depressive symptoms were assessed using the 10-item Center for Epidemiological Studies Depression Scale (CES-D-10; Cheng and Chan, 2005). Chronic diseases (including hypertension, dyslipidemia, diabetes or high blood sugar, cancer or malignant tumor, chronic lung diseases, liver disease, heart attack, coronary heart disease, angina, congestive heart failure, or other heart problems, stroke, kidney disease, stomach or other digestive diseases, arthritis or rheumatism, and asthma) were assessed *via* self-report. Each disease was given a point of 1 or 0 (presence vs. absence) and the possible sum score ranged from 0 to 12. Handgrip strength was objectively measured by a dynamometer, and the maximum of two hands was adopted.

### Statistical Analyses

Descriptive statistics were applied to show baseline characteristics of the enrolled participants. Mixed-effects models were developed to quantify the longitudinal associations of BMI, WC, change in BMI, and change in WC with cognitive decline. In our analysis, subjects and slope were set as random components to account for different rates of cognitive decline during the follow-up assessments. Separate models were fit for BMI and WC. First, we included terms of time, BMI, or WC, or BMI range or WC range, and covariates (age and sex), and their interaction with time. Then, we tested for sex differences in the relationships of BMI, or WC, or BMI range or WC range, with cognitive decline, by adding the interaction of sex, BMI or WC, and time, as well as interaction terms of sex, BMI range or WC range, and time. Finally, to examine the potential confounders of the associations of BMI and WC with cognitive decline, we adjusted for education, smoking status, drinking status, sleep duration, chronic diseases, depression, and grip strength. Additionally, we conducted longitudinal analyses using the categories of baseline BMI and WC with the normal and no central obesity as the referent, respectively. We also conducted longitudinal analyses using the categories of BMI change and WC change with the stable as the referent. Models also included the covariates mentioned above. All statistical analyses were performed using SAS version 9.4 (SAS Institute Inc., Cary, NC, USA). All *p*-values were two-tailed, and *p* < 0.05 were considered statistically significant.

## Results

A total of 3,035 older adults [mean (SD) age, 66.94 (5.43) years; 40.16% (*n* = 1,219) women] were enrolled in this study. The mean values of BMI and WC were 23.11 ± 3.91 kg/m^2^ and 84.56 ± 12.48 cm, respectively. The mean BMI range was 1.21 kg/m^2^, and the mean WC range was 5.80 cm. Both (1) BMI and WC and (2) BMI range and WC range were higher in women than in men. Within the sample, 1,658 subjects (54.63%) were classified as normal, 833 participants (27.45%) were classified as overweight, and 290 subjects (9.56%) were classified as obese. The proportion of no central obesity, central pre-obesity, and central obesity at baseline was 43.92, 15.85, and 40.23%, respectively. The mean global cognition *z*-score was −0.18, and the score was higher in men than in women. The characteristics of participants included in the baseline survey are presented by sex in Table 1.

**Table 1:** Baseline characteristics of participants.

**Characteristics^**a**^**	**Female**	**Male**	**Total**
	**(*N* = 1,219)**	**(*N* = 1,816)**	**(*N* = 3,035)**
Age, years	66.44 (5.30)	67.28 (5.50)	66.94 (5.43)
High education, *n* (%)^b^	58 (4.76%)	168 (9.25%)	226 (7.45%)
**Smoking status**, ***n*** **(%)**			
Never	1,087 (89.17%)	467 (25.72%)	1,554 (51.20%)
Former	35 (2.87%)	364 (20.04%)	399 (13.15%)
Current	97 (7.96%)	985 (54.24%)	1,082 (35.65%)
**Drinking status**, ***n*** **(%)**			
Never	984 (80.72%)	584 (32.16%)	1,568 (51.66%)
Former	82 (6.73%)	288 (6.73%)	370 (12.19%)
Current	153 (12.55%)	944 (51.98%)	1,097 (36.14%)
Sleep duration, hours	6.10 (1.91)	6.38 (1.82)	6.27 (1.86)
Chronic diseases, median (IQR)	1.00 (2.00)	1.00 (2.00)	1.00 (2.00)
Depression, n (%)	393 (32.24%)	405 (22.30%)	798 (26.29%)
Handgrip strength, kilogram	22.02 (6.46)	33.83 (7.76)	29.48 (8.97)
BMI, kg/m^2^	23.82 (4.13)	22.63 (3.67)	23.11 (3.91)
BMI range, median (IQR)	1.36 (1.46)	1.13 (1.25)	1.21 (1.36)
WC, cm	85.77 (13.00)	83.75 (12.05)	84.56 (12.48)
WC range, median (IQR)	6.60 (6.80)	5.50 (5.50)	5.80 (6.00)
**BMI groups**			
Underweight	94 (7.71%)	160 (8.81%)	254 (8.37%)
Normal	562 (46.10%)	1,096 (60.35%)	1,658 (54.63%)
Overweight	400 (32.81%)	433 (23.84%)	833 (27.45%)
Obese	163 (13.37%)	127 (6.99%)	290 (9.56%)
**WC groups**			
No central obesity	334 (27.40%)	999 (55.01%)	1,333 (43.92%)
Central pre-obesity	191 (15.67%)	290 (15.97%)	481 (15.85%)
Central obesity	694 (56.93%)	527 (29.02%)	1,221 (40.23%)
Global cognition, median (IQR)	−0.46 (1.41)	0.11 (1.42)	−0.18 (1.42)

a *Values are mean (SD), unless otherwise specified*.

b *High education: senior high school and above*.

*IQR, interquartile range; BMI, body mass index; WC, waist circumference*.

### BMI and Decline in Cognitive Function

Participants with a higher BMI had a slower rate of global cognition score decline (estimate = 0.0107; SE = 0.0024; *p* < 0.0001), whereas greater BMI variability was associated with a faster rate of global cognition score decline (estimate = −0.0365; SE = 0.0116; *p* = 0.0017); and there were similar patterns of findings for memory. Higher BMI was related to a slower rate of decline in orientation and attention score (estimate = 0.0070; SE = 0.0026; *p* = 0.0089). However, data showed no significant three-way interactions between sex, BMI, and time, or between sex, BMI range, and time for the decline in global cognition score or any subdomains (Table 2). In addition, global cognition score in individual with BMI-defined overweight (estimate = 0.0094; SE = 0.0043; *p* = 0.0298) declined slower than in the reference group (normal baseline BMI group) after adjusting for a number of covariates (Table 4). Compared with participants with stable weight, participants with large weight gain had a significantly accelerated rate of global cognition score decline (estimate = −0.0266; SE = 0.0074; *p* = 0.0003), while no significantly accelerated rate of global cognition score decline was detected in those with large weight loss (**Table 5**).

**Table 2:** The association between body mass index (BMI), BMI range, and decline among global cognition and cognitive subdomains (SD per year).

**Variable**	**Global cognition estimate (SE), *p*-value**	**Memory estimate (SE), *p*-value**	**Orientation and attention estimate (SE), *p*-value**	**Visuospatial ability estimate (SE), *p*-value**
Age	0.0024 (0.0015), 0.1048	0.0015 (0.0017), 0.3745	0.0020 (0.0016), 0.2176	0.0015 (0.0018), 0.4047
Sex (male)	−0.0062 (0.0189), 0.7415	0.0032 (0.0210), 0.8788	−0.0020 (0.0204), 0.9231	−0.0257 (0.0230), 0.2648
Time	−0.0587 (0.0242), 0.0051*	−0.0533 (0.0270), 0.0486*	−0.0358 (0.1345), 0.4236	−0.0491 (0.0295), 0.0965
BMI	−0.0007 (0.0023), 0.7779	−0.0018 (0.0026), 0.5030	0.0005 (0.0025), 0.8526	−0.0011 (0.0029), 0.6940
BMI range	0.0004 (0.0037), 0.9180	0.0003 (00.9402	0.0002 (0.0040), 0.9632	0.0005 (0.0045), 0.9084
BMI × time	0.0107 (0.0024), <0.0001*	0.0097 (0.0026), 0.0002*	0.0070 (0.0026), 0.0089*	0.0043 (0.0028), 0.1350
BMI range × time	−0.0365 (0.0116), 0.0017*	−0.0481 (0.0126), 0.0001*	−0.0050 (0.0127), 0.6949	−0.0183 (0.0138), 0.1840
Sex × BMI × time	−0.0001(0.0003), 0.7390	<0.0001 (0.0003), 0.9080	−0.0002 (0.0004), 0.6134	−0.0001 (0.0004), 0.8437
Sex × BMI range × time	0.0028 (0.0017), 0.0966	0.0023 (0.0018), 0.2031	0.0015 (0.0018), 0.4218	0.0007 (0.0020), 0.7305

Four separate mixed-effects models, each adjusted for age, education, smoking status, drinking status, sleep duration, chronic diseases, depression, and handgrip strength. BMI, body mass index; ^*^p < 0.05.

### WC and Decline in Cognitive Function

Greater WC was significantly related to slower memory score decline (estimate = 0.0014; SE = 0.0007; *p* = 0.0493), orientation and attention score decline (estimate = 0.0014; SE = 0.0007; *p* = 0.0480), and global cognition score decline (estimate = 0.0019; SE = 0.0006; *p* = 0.0037). The association between person-specific WC range and cognition score decline was not statistically significant for any of the cognitive outcomes. Furthermore, sex did not modify the relationship of WC or WC range with cognition score decline in any cognitive outcomes (Table 3). We also found that, compared with participants under stable WC status, participant with large loss was associated with faster rate of global cognition score decline (estimate = −0.0668; SE = 0.0329; *p* = 0.0426; Table 5).

**Table 3:** The association between waist circumference (WC), WC range, and decline among global cognition and cognitive subdomains (SD per year).

**Variable**	**Global cognition estimate (SE), *p*-value**	**Memory estimate (SE), *p*-value**	**Orientation and attention estimate (SE), *p*-value**	**Visuospatial ability estimate (SE), *p*-value**
Age	0.0027 (0.0015), 0.0757	0.0019 (0.0017), 0.2635	0.0021 (0.0017), 0.2017	0.0016 (0.0019), 0.3974
Sex (male)	−0.0011 (0.0189), 0.9522	0.0071 (0.0213), 0.7408	0.0033 (0.0207), 0.8737	−0.0255 (0.0233), 0.2743
Time	−0.0267 (0.0300), 0.3725	−0.0313 (0.0334), 0.3146	−0.0078 (0.0328), 0.8002	−0.0248 (0.0365), 0.5089
WC	−0.0001 (0.0007), 0.8758	−0.0004 (0.0008), 0.5906	0.0002 (0.0008), 0.8049	0.0003 (0.0009), 0.7282
WC range	−0.0001 (0.0006), 0.8879	−0.0005 (0.0009), 0.6132	0.0003 (0.0009), 0.7113	−0.0002 (0.0009), 0.8324
WC × time	0.0019 (0.0006), 0.0037*	0.0014 (0.0007), 0.0493*	0.0014 (0.0007), 0.0480*	0.0009 (0.0007), 0.2491
WC range × time	0.0016 (0.0020), 0.4319	0.0021 (0.0023), 0.3575	0.0017 (0.0022), 0.4566	−0.0030 (0.0025), 0.2345
Sex × WC × time	0.0001(0.0001), 0.9931	<0.0001 (0.0001), 0.9740	−0.0001 (<0.0001), 0.8888	−0.0001 (0.0001), 0.6106
Sex × WC range × time	0.0001(0.0003), 0.7094	0.0004 (0.0004), 0.2424	−0.0005 (0.0003), 0.1277	0.0005 (0.0003), 0.1804

Four separate mixed-effects models, each adjusted for age, education, smoking status, drinking status, sleep duration, chronic diseases, depression, and handgrip strength. WC, waist circumference; ^*^p < 0.05.

**Table 4:** The association between global cognitive decline (SD per year) comparing quartiles of baseline BMI and WC.

	**Participants**	**β**	**SE**	***p*-value**
**BMI groups**				
Underweight	254	−0.0025	0.0070	0.7184
Normal	1,658	Reference	—	—
Overweight	833	0.0094	0.0043	0.0298*
Obese	290	0.0058	0.0065	0.3724
**WC groups**				
No central obesity	1,333	Reference	—	—
Central pre-obesity	481	0.0037	0.0054	0.4955
Central obesity	1,221	0.0056	0.0042	0.1797

Adjusted for age, education, smoking status, drinking status, sleep duration, chronic diseases, depression, and handgrip strength. BMI, body mass index; WC, waist circumference; *p < 0.05.

**Table 5:** The association between global cognitive decline (SD per year) comparing quartiles of BMI and WC change.

	**Participants**	**β**	**SE**	***p*-value**
**BMI change groups**				
Large loss	248	−0.0090	0.0076	0.2356
Small loss	403	−0.0077	0.0058	0.1836
Stable	1,565	Reference	—	—
Small gain	556	0.0032	0.0050	0.5262
Large gain	263	−0.0266	0.0074	0.0003*
**WC change groups**				
Large loss	365	−0.0668	0.0329	0.0426*
Small loss	409	−0.0278	0.0276	0.3139
Stable	1,236	Reference	—	—
Small gain	600	−0.0003	0.0217	0.9909
Large gain	425	0.0291	0.0285	0.3071

Adjusted for age, education, smoking status, drinking status, sleep duration, chronic diseases, depression, and handgrip strength. BMI, body mass index; WC, waist circumference; *p < 0.05.

## Discussion

This study adds to the emerging literature concerning the relationship between BMI, WC, and cognitive decline over a 7-year follow-up using a nationally representative sample of Chinese older adults. The results showed that higher BMI and WC were associated with slower cognitive decline, and greater BMI variability was related to a faster rate of cognitive decline in late life. No difference in these associations was found by sex. We observed a slower cognitive decline among participants who were overweight. In addition, participants with large weight gain and large WC loss both were associated with faster cognitive decline.

Previous research findings on BMI, WC, and late-life cognitive decline among older adults are mixed, with results showing both detrimental and protective effects of higher BMI and WC on the decline in cognitive function. Several studies found that participants with overweight and obesity defined by BMI had better cognitive scores, while those with underweight had lower performance (Kerwin et al., 2010; Coin et al., 2012; Tolppanen et al., 2014). Other studies indicated that higher later-life BMI was related to a slower rate of cognitive decline (Atti et al., 2008; Luchsinger et al., 2013; Kim et al., 2016, 2020; Deckers et al., 2017; Rodriguez-Fernandez et al., 2017; Arvanitakis et al., 2018). Li et al. found that the association of BMI and cognitive function was inversely U-shaped among Chinese older adults (Li et al., 2020). Another study in China, involving 1,100 participants aged 60–98 years (mean age = 79 years), found that overweight defined by BMI was significantly related to a lower risk of cognitive impairment (Hou et al., 2019). These results were consistent with findings in our study that BMI-defined overweight has an apparent protective effect for cognitive decline among Chinese older adults. Another finding of this study is that greater WC is a significant protective factor against cognitive decline. The Cardiovascular Health study, with subjects having a mean age over 73 years, indicated that a higher WC is related to slower cognitive decline in Americans (Luchsinger et al., 2013). In contrast, a cohort study conducted in America reported that larger WC in late life was associated with faster cognitive decline (West et al., 2017). Future studies are needed to establish a clear relationship between WC and cognitive change in late life.

Several plausible biological pathways may help explain the protective effect of high BMI and WC for cognitive decline. First, the hormone leptin, secreted mainly from the adipose tissue, might contribute to regulate the hippocampal synaptic plasticity and to slow the cognitive decline (Harvey et al., 2006), as it is established that the hormone leptin contributes to enhance memory performance in rodents (Oomura et al., 2006). Second, individuals with low BMI may reflect nutritional deficiencies that aggravate neurodegeneration, such as low vitamin B levels with the secondary elevation of homocysteine levels (Coin et al., 2012), or have low levels of important brain trophic factors (e.g., brain-derived neurotrophic factor) that deteriorate the development of neurodegenerative disease (Zuccato and Cattaneo, 2009). Third, the relationship may be mediated by serum urate, which is positively associated with BMI and may suppress the process of neurodegenerative disease by acting as antioxidants in the brain (Chen et al., 2009). In addition, this may be due to the “survival bias effect,” in which individuals who are with high BMI might be healthier among their peers in late life. A previous study stated that older persons who were overweight had a lower mortality risk than those who were of normal weight (Dahl et al., 2013).

The dynamic changes in BMI and WC may influence cognitive performance. We found a positive association between a greater BMI variability and a faster rate of cognitive decline, which was in consistent with previous findings. A prospective study involving 671 older adults in a 6-year follow-up reported that a greater BMI variability was associated with a faster cognitive decline (Aiken-Morgan et al., 2020). We also found that Chinese older adults with both large weight gain and large WC loss were associated with faster cognitive decline. A nationally representative American cohort indicated that a BMI decrease and a WC increase >10% over a 3-year follow-up were related to an increased risk of cognitive decline among older adults (Rodriguez-Fernandez et al., 2017), and a recent large cohort study in China reported that large weight loss was associated with an increased risk of cognitive impairment (Wu et al., 2021). Losing weight was found to be associated with worse cognitive performance or greater risk of cognitive impairments in later life (Atti et al., 2008; Hughes et al., 2009; Tolppanen et al., 2014; West et al., 2017). Significant weight loss was identified as a predictor of impending dementia (Thomas, 2007). That might be due to sarcopenia (Grundman, 2005) and hormonal factors (Bagger et al., 2004) or might be a sign of ill health for older adults (Dahl et al., 2013), in which greater BMI variabilities are suggestive of systemic breakdown (Arnold et al., 2010). The findings have demonstrated that it may be beneficial to evaluate cognitive function in elderly individuals with greater weight variability.

In addition, the effect of BMI and WC on the decline in cognitive function may be driven by sex differences. Compared with men, women exhibited a greater weight on average in late life, and women also tend to have higher weight fluctuation (Walsemann and Ailshire, 2011; Aiken-Morgan et al., 2020). This is confirmed in this study, as women had greater BMI (23.82 vs. 22.63), WC (85.77 vs. 83.75), BMI variability (1.36 vs. 1.13), and WC variability (6.60 vs. 5.50) than men. At present, only one prospective study, involving 671 older adults in a 3-year follow-up, reported the potential confounding role of sex, and no modification effect was found on the association of obesity with cognitive decline (Aiken-Morgan et al., 2020), which is in agreement with our study. Additional large prospective studies are needed to explore sex differences in the effects of BMI and WC on cognitive change in the older population.

### Strengths and Limitations

Our study was based on a nationally representative longitudinal dataset. To the best of our knowledge, this is the first cohort study to assess the associations of BMI, WC, change in BMI, and change in WC with cognitive decline among older adults in China where the aging-related diseases are expanding rapidly. Furthermore, two clinically available measures of obesity (i.e., BMI and WC) were objectively measured at three examinations over 7 years. This study does have some limitations. First, although we have adjusted the data for multiple confounding factors, such as education, smoking status, drinking status, sleep duration, chronic diseases, depression, and handgrip strength, it is possible that residual factors that were not captured might modify or mediate the relationship between obesity and cognitive function. Second, we recognize that this study was only based on a sample of Chinese older adults, making it difficult to interpret whether our findings are applicable to older adults in other countries. Third, as this study is observational, we cannot determine causality. Novel statistical approaches (e.g., Mendelian randomization) should be considered to overcome the limitations of traditional observational studies and to elucidate the influences of obesity on cognition in the future.

## Conclusion

Higher BMI and higher WC have a protective effect against cognitive decline in late life, and the greater BMI variability is related to the faster rate of cognitive decline. Overweight was associated with a slower cognitive decline, and both large weight gain and large WC loss were associated with faster cognitive decline. From a clinical perspective, monitoring weight status for early detection and providing weight management advice may bring benefits for dementia prevention.

## Data Availability Statement

The data presented in this study are available from the corresponding author upon reasonable request. The CHARLS questionnaires are available at http://charls.pku.edu.cn/pages/data/111/zh-cn.html.

## Author Contributions

FL, JF, and RL designed the study. FL, JF, NQ, and YW acquired and collected the data. FL, JF, and YX analyzed the data. FL and JF drafted the manuscript. JBM and XZ revised the manuscript. RL critically reviewed and revised the manuscript. All authors contributed to this article and approved the submitted version.

## Conflict of Interest

The authors declare that the research was conducted in the absence of any commercial or financial relationships that could be construed as a potential conflict of interest.

## Publisher's Note

All claims expressed in this article are solely those of the authors and do not necessarily represent those of their affiliated organizations, or those of the publisher, the editors and the reviewers. Any product that may be evaluated in this article, or claim that may be made by its manufacturer, is not guaranteed or endorsed by the publisher.

## Footnotes

^1^P.D. World Population Prospects: The 2017 Revision (online).
